# Intra-articular adhesions of the temporomandibular joint: Relation between arthroscopic findings and clinical symptoms

**DOI:** 10.1186/1471-2474-10-70

**Published:** 2009-06-17

**Authors:** ShanYong Zhang, XiuMing Li u, Chi Yang, XieYi Cai, MinJie Chen, Majd S Haddad, Bai Yun, ZhuoZhi Chen

**Affiliations:** 1 Department of Oral and Maxillofacial Surgery, Ninth People's Hospital, School of Medicine, Shanghai Jiao Tong University, No. 639, Zhi Zao Ju Rd, 200011, Shanghai, PR China; 2 College of Dentistry, University of Iowa, Iowa 52242 USA

## Abstract

**Background:**

Intra-articular adhesion (IA) is one of the important pathologic signs of intracapsular temporomandibular joint (TMJ) diseases, but this factor has been rarely described with respect to its arthroscopic characteristics and histology. The purpose of this study was to describe the incidence and distribution of IA in patients with internal derangement (ID) and to investigate the correlation between adhesions and the clinical symptoms of patients with ID of TMJ with closed-lock.

**Methods:**

A retrospective analysis was conducted of 1822 TMJs with ID that were refractory to nonsurgical treatments and underwent arthroscopic surgery between May 2001 and June 2008 in our department. Clinical findings were assessed on the basis of mandibular range of motion, patients' age and locking duration at the initial visit. ID stages were judged according to the Wilkes and Bronstein classification based on clinical symptoms and pre-operative magnetic resonance imaging. 1506 patients (1822 joints) with ID were divided into an adhesion group (486 patients) and a non-adhesion group (1020 patients). The associations between the two groups with respect to interincisal opening, clicking duration, locking duration and patients' age were statistically analyzed using a *t*-test.

**Results:**

Arthroscopy confirmed occurrences of adhesion in 28.76% of the joints (524 joints out of a total of 1822). Grade 1 adhesion was found in 68.89% of those cases; grade 2 in 20.61%; grade 3 in 4.58%; and grade 4 in 5.92%. The percentages of instances of adhesion in different stages were as follows: 13.89% of the joints in Stage II had adhesion, 25.47% in Stage III, 37.99% in Stage IV, and 40.37% in Stage V. There were statistically significant differences for patients' age (*t = 10.41, P < 0.001*), interincisal opening (*t = 9.54, P < 0.001*), paining duration (*t = 3.66, P < 0.001*) and locking duration (*t = 3.89, P < 0.001*) between the two groups, while no statistically significant difference was found for clicking duration (*t = 1.08, P > 0.05*).

**Conclusion:**

The arthroscopic findings confirmed that the incidence ratio of adhesion was high and occurred predominantly with older patients with longer locking duration and less interincisal opening. As the stage of ID increased, the adhesion grade rose.

## Background

Murakami and Segami [1] elucidated the nature and pathogenesis of adhesion formation, described its arthroscopic characteristics and the correlation between adhesions and the clinical subjective symptoms of patients with internal derangement of the temporomandibular joint (TMJ) with closed-lock, and suggested the frequent occurrence of intra-articular adhesion (IA) of TMJ. However, the most common method of imaging used to diagnose adhesions certainly has its limitations. Plain film arthrography (PFA) is restricted because of the projection angle and its radiation capability. Although magnetic resonance imaging (MRI) is certainly a less invasive procedure with which to image TMJ disc, the diagnostic accuracy of MRI for intra-articular adhesions was poor and IA could only be detected on T2 weighted-images with existing synovial fluid. Magnetic resonance arthrography (MRAr) has a relatively higher accuracy, but the high cost and the required special skills constrain its use [2-5]. Arthroscopy can examine adhesions directly and observe various adhesions in the upper compartment of TMJ. Although it is a costly procedure, arthroscopy is also, particularly if done in the operating room under general anesthesia. The advantage of arthroscopy is that it is more accurate in determining the location and type of adhesions. If such intra-articular diseases are detected, they could be removed by performing various operations at the same time [6-10]. Therefore arthroscopy is considered as a better way to diagnose and treat IA. In order to provide guidance for clinical treatment, in this study we investigated the incidence ratio of the TMJ IA and explored the correlation between two groups with regard to locking duration, interincisal opening, paining duration and patients' age.

## Methods

### Patients

Consecutive 1822 TMJs of 1506 patients with unilateral (1189 patients) or bilateral (317 patients) internal derangement (ID) of the TMJ who visited the TMJ clinic at the Ninth People's Hospital, affiliated with the Shanghai JiaoTong University, between May 2001 and June 2008. The patients whose ID stages varied from II~V were diagnosed in accordance with the clinical and imaging standard of Wilkes and Bronstein [11,12]. There were 281 men and 1225 women with a mean age of 29.79 years (range 12–73). The mean duration of TMJ closed-lock before arthroscopy was 6.97 months, ranging from 0.5 to 96 months. Those patients had experienced severe TMJ pain and/or disturbance at mouth opening and were refractory following nonsurgical treatments aimed at alleviating their symptoms. Patients with previous TMJ surgery, systemic inflammatory joint disease, facial growth disturbances, direct trauma or fractures of facial bones, condylar hypoplasias, or tumor were excluded.

Clinical examination and MRI were performed, and signs and symptoms were recorded at the initial visit. MRI was performed on all patients by using a 1.5-T MRI scanner (General Electric, Milwaukee, Wis) with bilateral 3-inch dual-surface coils, as described previously [3]. Arthroscopic surgeries were performed by one of the authors (C. Yang) after the MRI showed disc displacement with or without reduction which had been treated with nonsurgical modalities for some time. The study was approved by the Human Research Ethics Committee at The Ninth People's Hospital's and the Standing Committee on Ethics in Research Involving Humans of Shanghai Jiao Tong University. All participants gave written informed consent.

### Instruments and equipment

The equipments included an arthroscopy system (Stryker, San Jose, CA) and a video system (Sony Company, Japan). A 2.4 mm arthroscope with a 2.7 mm outer protective cannula was used for diagnostic arthroscopy of the superior joint space of the TMJ.

### Arthroscopic surgery

**Figure 1:**
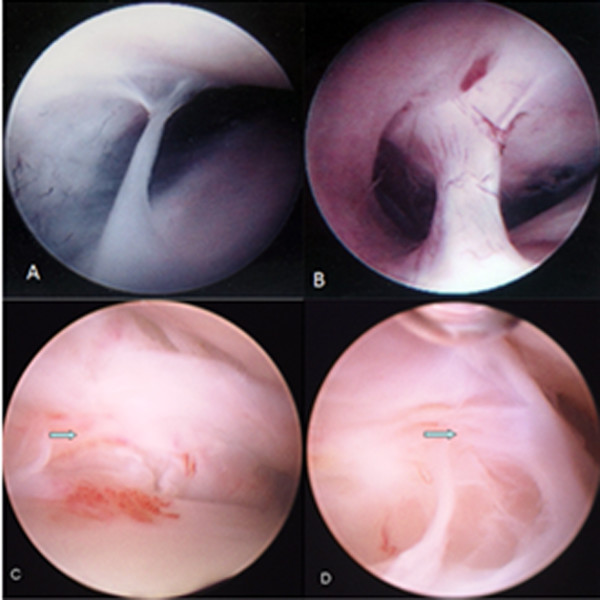
**Different adhesions under arthroscopy**. A. Arthroscopic view of grade 1 adhesion; B. Arthroscopic view of grade 2 adhesion; C. Arthroscopic view of grade 3 adhesion; D. Arthroscopic view of grade 4 adhesion.

Arthroscopic examinations and treatments were performed by the same specialist. Patients were under local anesthesia during the examination and treatment. After upper joint distention was achieved with 2–3 ml of 2% lidocaine, the space was entered with a sharp trocar, 2.4 mm in diameter, protected by 2.7 mm outer cannula. Once the capsule was entered (the surgeon confirmed entry by the presence of fluid return in the cannula), a blunt obturator was used to replace the sharp trocar. The obturator provided further confirmation of correct placement and allowed for gentle palpation of the fibrocartilage. Next, the 2.4 mm arthroscope was inserted, and an initial diagnostic sweep from the posterior syonvial pouch to the anterior syonvial pouch was performed. Capsular distention and intermittent lavage with Ringer lactate irrigation were maintained through an open irrigation system. In the upper compartment, the structures including the posterior attachment of the disc, the disc itself, the fibrocartilage of the glenoid fossa, and the synovium were examined. Detection of the intra-articular adhesions also allowed for the simultaneous application of a particular treatment. The shape, location, and degree of adhesions were recorded postoperatively by the assistant with nine parts of the TMJ upper compartment (Figure 1) [13]. The upper joint compartment was first divided into 3 regions, the anterior recess as region I, the intermediate space as region II, and the posterior recess as region III. Then, each region was further divided into 3 parts from lateral to medial.

Based on the classification of Professor Yang [14], adhesions were divided into 4 grades under arthroscopy as shown in Figure 1. Grade 1 was identified as a filmy or silk-like adhesion connecting the intersynovial structures from the roof to the bottom structure. This pathology was observed both in the anterior and the posterior synovial pouch or recess, and usually in the medial capsular area (Figure 1A). Grade 2 was commonly observed in the anterolateral aspect of the upper joint compartment. As this broad adhesion is frequently formed at the anterior boundaries of the upper joint compartment, distinguishing the real capsule from this structure is not easy. Under precise and appropriate observation and palpation in the anterior recess, the adhesion surface with irregular distribution of capillary networks was revealed (Figure 1B). Grade 3 was defined as band-like adhesion connecting the glenoid fossa and the posterior disc band. It was usually detected in the intermediate space between the articular eminence and the articular disc or the posterior attachment. It could lead to moderate mouth-opening limitation (Figure 1C). Grade 4 is defined as an extensive adhesion, being formed in more than 3 parts of the joint compartment. This type of adhesion could also lead to severe mouth-opening limitation (Figure 1D). After operation, the shape, position and degree of adhesions were recorded by the assistant according to their coincident opinions with the disk being divided into nine parts [13].

### Statistical analysis

The data were processed using the Statistical Package for the Social Sciences (SPSS version 13.0) software. The intergroup differences between the adhesion group and non-adhesion group were compared by *a t*-test, and the correlation between the different grades of adhesion and variable ID stages were processed by a X^*2*^-test, as *p *values less than 0.05 were considered significant.

## Results

### Relationship between adhesion grades and ID stages

**Figure 2:**
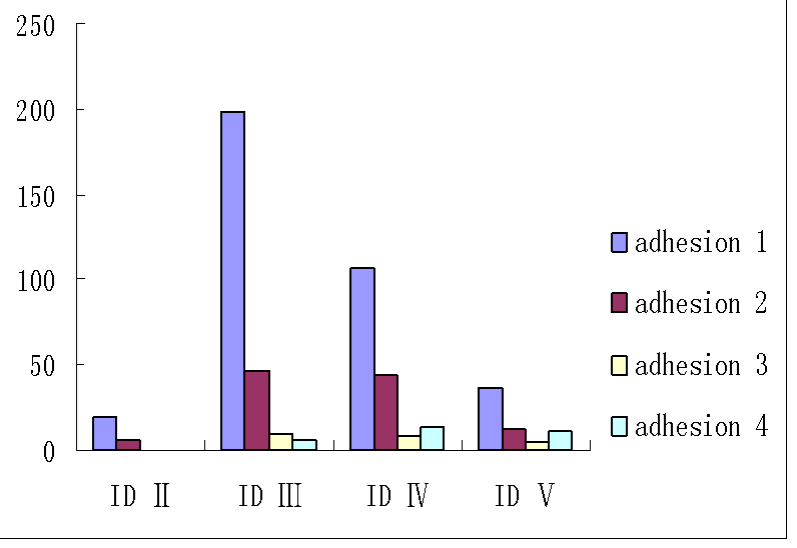
**The relationship between the adhesion of different grades and variable ID stages**.

Arthroscopic surgery confirmed that 524 joints out of 1822 TMJs in 486 patients had adhesions, which accounted for 28.76%. The relationship between adhesion grade and ID stage is presented in Figure 2. The percentage of grade 1 adhesions was 68.89% (361/524), 20.61% (108/524) were grade 2, 4.58% (24/524) were grade 3, 5.92% (31/524) were grade 4. Twenty-five out of 180 TMJs (13.89%) which were in stage II of ID had adhesions. The number of adhesions among the 1021 TMJs which were in stage III of ID was 260 (25.47%). One hundred and seventy-four (37.99%) of the instances of adhesion were observed with the 458 TMJs which were in stage IV of ID. Finally, adhesions were detected in 65 from the total of 161 TMJs which were in stage V of ID (40.37%). There was significant correlation between the different grades of adhesion and the variable ID stages (X^2 ^= 46.54, P < 0.01)

### Distribution of adhesions in upper joint compartment

**Figure 3:**
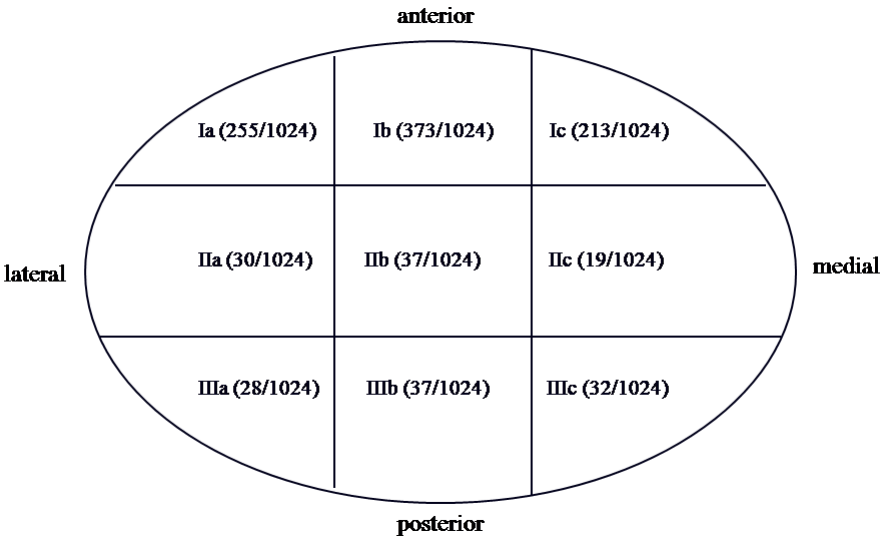
**Distribution of adhesions in the upper joint compartment**.

As shown in Figure 3, there were 4716 parts in the 524 TMJs with adhesions, and 1024 of those parts had adhesions. As regards adhesion location, 841 (82.13%) were in region I, 86 (8.40%) were in region II, and 97 (9.47%) were in region III. Two hundred and fifty-five (24.90%) out of the total 1024 parts with adhesion were in I a, 373 (36.43%) were in I b, 213 (20.80%) were in I c, 30 (2.93%) were in II a, 37 (3.61%) were in II b, 19 (1.86%) were in IIc, 28 (2.73%) were in IIIa, 37 (3.61%) were in IIIb, and 32 (3.13%) were in IIIc.

### Clinical symptom comparison between the two groups

The arthroscopy confirmed that 486 patients (524 joints) were in an adhesion group and 1020 patients (1298 joints) were in a non-adhesion group. Comparative analysis of the obtained data from the two groups was performed with regard to patients' age, clicking duration, locking duration, paining duration and maximum interincisal opening as shown in Table 1. There were significant differences between the adhesion group and the non-adhesion group for all of the items (P < 0.001) stated above with the exception of clicking duration.

**Table 1:** Comparison of patients'age, clicking duration, locking duration, paining duration and interincisal opening between the adhesion group and the non-adhesion group

Item	Adhesion group (*n *= 490)	Non-adhesion group (*n *= 1230)	*t*	P*
Age (years)	35.03 ± 14.10	27.33 ± 11.78	10.41	.000*
Clicking duration (month)	18.91 ± 32.18	20.59 ± 27.85	1.08	0.281
Locking duration (month)	6.97 ± 8.38	5.42 ± 4.34	3.89	.000*
Paining duration (month)	4.61 ± 4.80	3.71 ± 4.03	3.66	.000*
IO pre-operation (mm)	24.33 ± 6.82	28.38 ± 8.18	9.54	.000*

Note: *P < 0.001; IO: Interincisal opening

## Discussion

No unified consensus exists as regards the classification and grades of TMJ IA. Murakami [1] has divided the adhesions into three types according to their shapes, namely film-like adhesion, band-like adhesion, and pseudowall-like adhesion. Other researchers have proposed a 6-type division of IAs based on their shape and location, or a 10-grade severity-based classification [14,15]. For the purposes of our study, we divided IAs into grades 1~4 according to the criteria established by Professor Yang and based on the main indexes of shape, size and location of the adhesion, besides TMJ function. The assignment of higher adhesion grades was correlated with a higher severity in the degree of mouth opening limitation. For example, if the band-like adhesion was located in the intermediate space, thus causing severe limitation on mouth opening, it was defined as grade 3. Using arthroscopy as the golden standard, we have compared the detection of the adhesion in 27 consecutive patients (33 joints) by MRI, and the article will be published soon [3]. So we will not do further discussion in this paper.

An earlier study by Murakami [16] reported the occurrence of IAs in 91.20% of his total of 68 patients with TMJ ID, which was much higher than our results. What might be accountable for that discrepancy is the difference in the ID stages of the patients. Our study indicated that stage III accounted for 56.04% of all cases, and stage IV for 25.14%, while in Murakami's study, stage IV and V were the most and the percentage was 44.12% totally. Pseudowall adhesion was the predominant pathology (80.9%, or 55 out of 68 joints) in Murakami's report. However, our statistical data indicated that the most common adhesion was grade 1 with 68.89%, followed by grade 2 with 20.61%, and grade 4 with 5.92%. Grade 3 was the least common adhesion and the percentage was only 4.58%.

The pathogenesis and etiology of IA have not been elucidated completely. There are different hypotheses on the mechanism of the etiology and the formation of adhesions. Kaminishi and Davis [17] hypothesized on the formation of adhesions in the TMJ based on the orthopedic literature. One of the theories claims that synovitis causes fibrin deposition, which decreases joint lubrication. The resultant suction cup effect and immobilized joint causes the fibrin deposition to continue, with the formation of fibrous adhesions. Another theory ascribes the formation of adhesions to hematomas in the synovial membrane which attract fibroblasts and fibrocytes to that area. The healing process is associated with the subsequent formation of scar tissue on the existing fibrous capsular wall.

In this study, we found that the adhesion in region I (anterior upper recess) was predominant accounting for 82.13% of adhesion occurrences. Among those, 61.33% were located in the exterior-middle parts (Ia and Ib). However, adhesions in region II and III constituted only 9.47% of the total number of adhesions. Similar conclusions have also been drawn by other studies [1,18]. A possible explanation to our findings could be the fact that the anterior upper recess is located in the lowest position of the upper joint compartment. This situation occurred more frequently if anterior disc displacement without reduction was present, especially if the disc was folded into a V or U-shape. The possible mechanism responsible for this might involve mechanical squeezing of the synovium in the anterior recess and the posterior attachment due to anterior disc displacement without reduction resulting in folding and fusion of the synovial membrane combined with vessels congestion and exudation in the plicate synovial membrane in cases of inflammation. The inflammation exudate accumulates in the anterior recess and the result is formation of an adhesion.

The patients in this study with ID stages II~V were divided into an adhesion group and a non-adhesion group. We investigated the correlation between patients' age, clicking duration, locking duration, paining duration and maximum interincisal opening between the two groups. From the statistical data, we knew that the patients' age and the locking duration in the adhesion group were higher than in the non-adhesion group, and the maximum interincisal opening before operation was bigger in the non-adhesion group than in the adhesion group. It was illustrated that longer locking duration and older age are associated with a higher possibility for adhesion formation as well as the occurrence of higher ID stages and the higher adhesion grades. Therefore, it is our opinion that patients with ID should be treated as soon as possible. A number of methods should be used to minimize the duration of the mouth opening limitation and, accordingly, to minimize the severity of the adhesion and to obtain satisfactory therapeutic effect.

## Conclusion

Various IAs are frequently detected in the upper joint compartment in patients with ID of TMJ. Adhesion grades may be described as grade 1 to grade 4, and adhesion incidence has a tendency to increase with the duration of symptoms and patients' age. The relationship between clinical signs and symptoms is reflected more distinctly in the degree of distribution of adhesions than in each regional level. Correlations between subjective symptoms might be considered as a concomitant disease in cases of intra-articular TMJ pathology.

## Competing interests

The authors declare that they have no competing interests.

## Authors' contributions

SYZand CYparticipated in the design of the study. SYZparticipated in the acquisition of data and wrote the paper. *All authors *carried out the arthroscopic operation, and recorded the patients' data. XMLperformed the statistical analysis and interpretation of data, and drafted the manuscript. XYC, MJC, BY, and ZZCparticipated in the analysis and interpretation of data, and reviewed the manuscript. MSHparticipated in the acquisition of data and corrected the English grammar. All authors read and approved the final manuscript.

## Pre-publication history

The pre-publication history for this paper can be accessed here:


